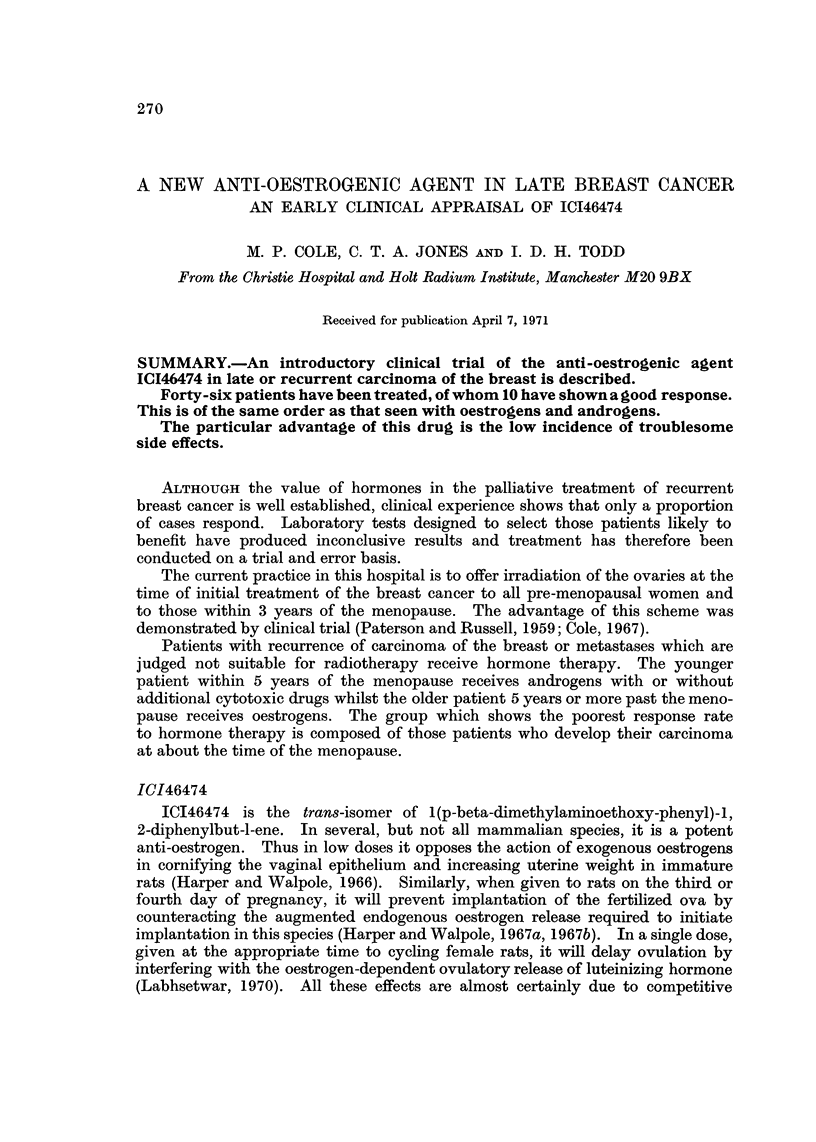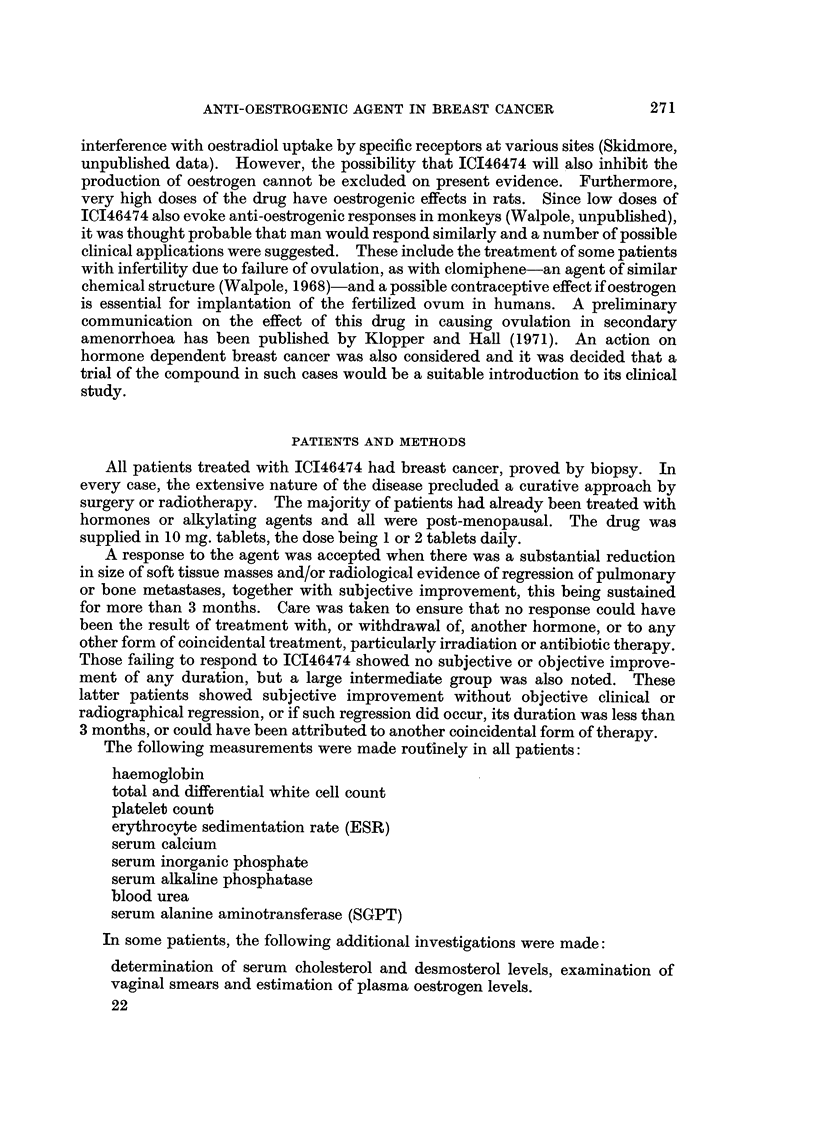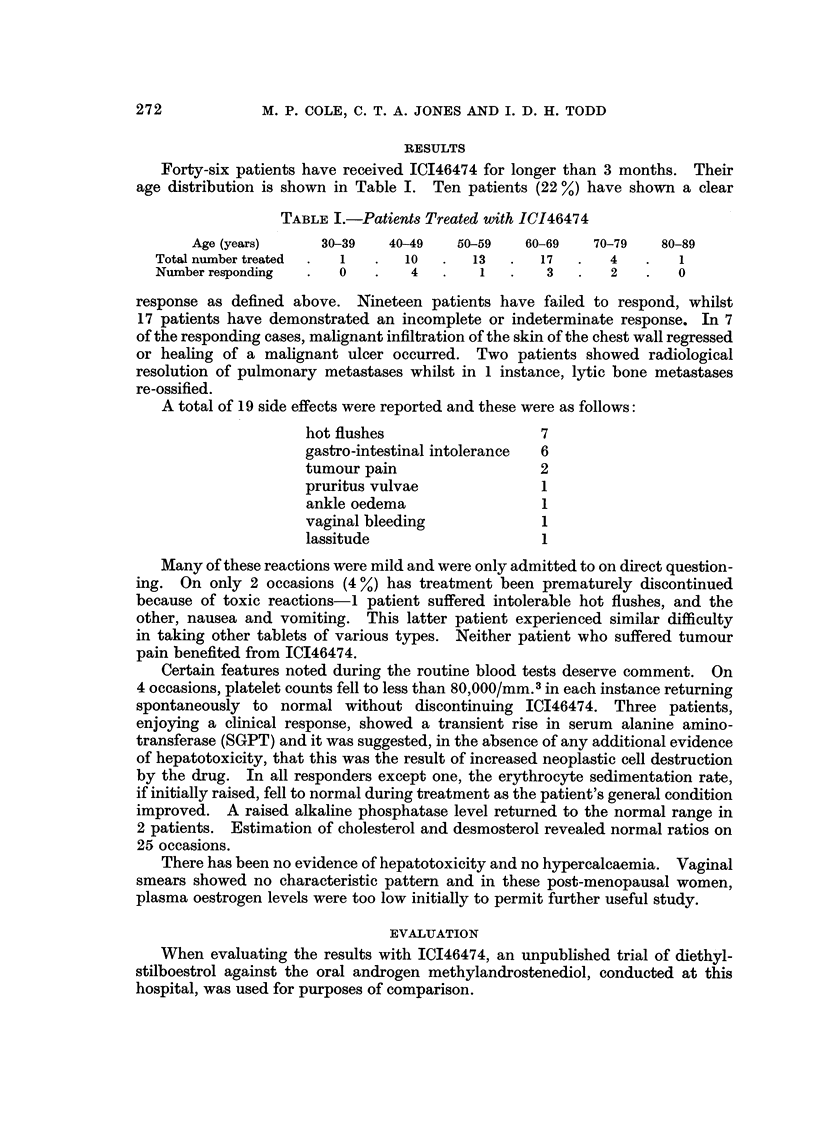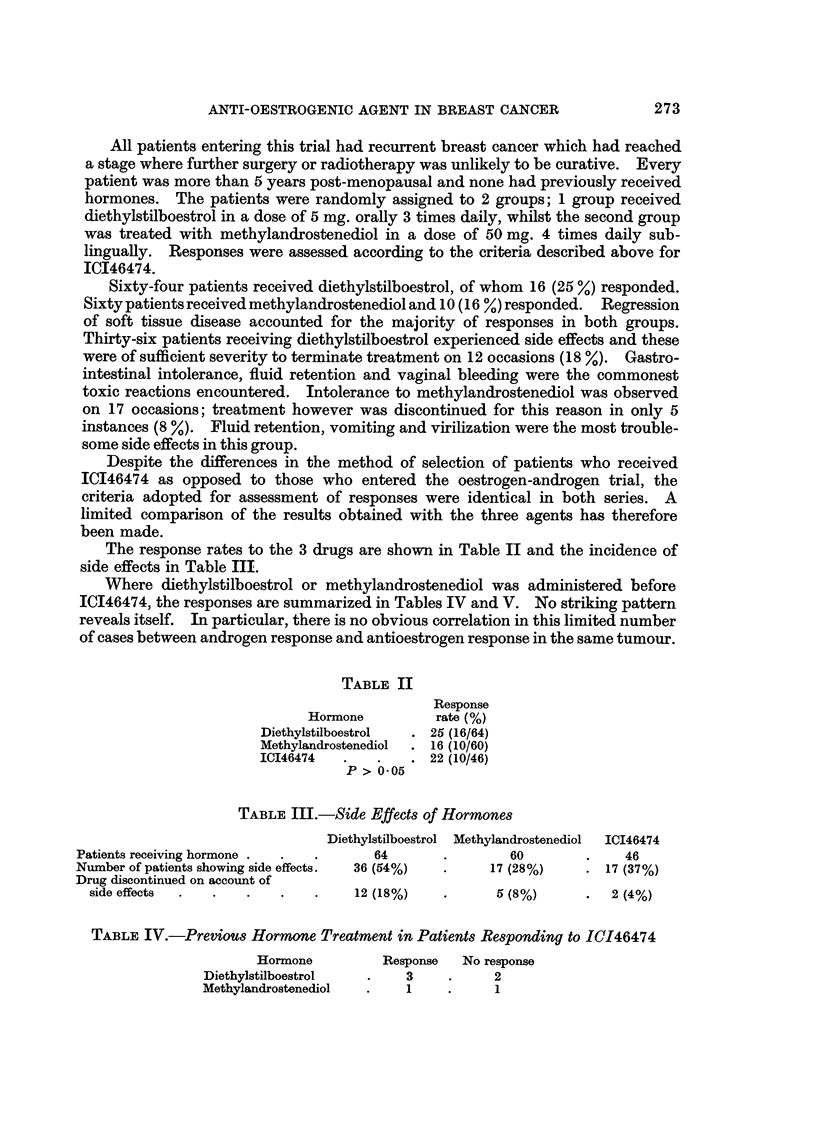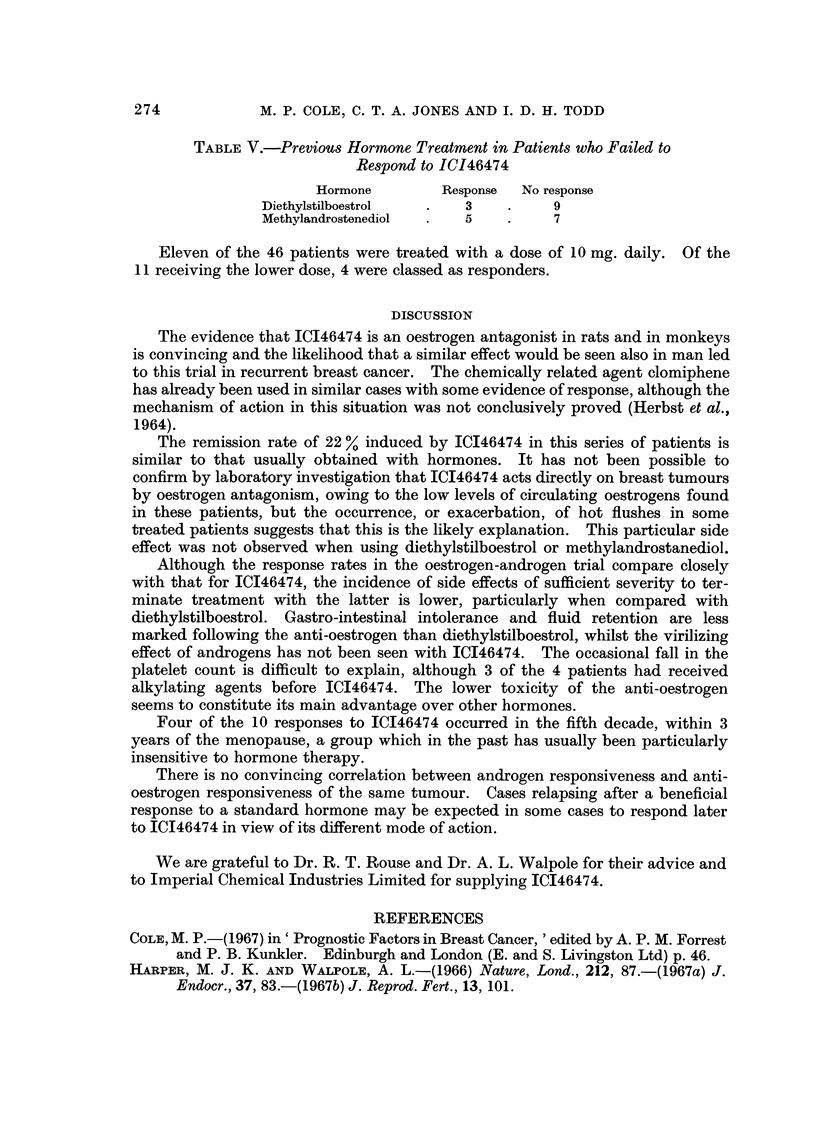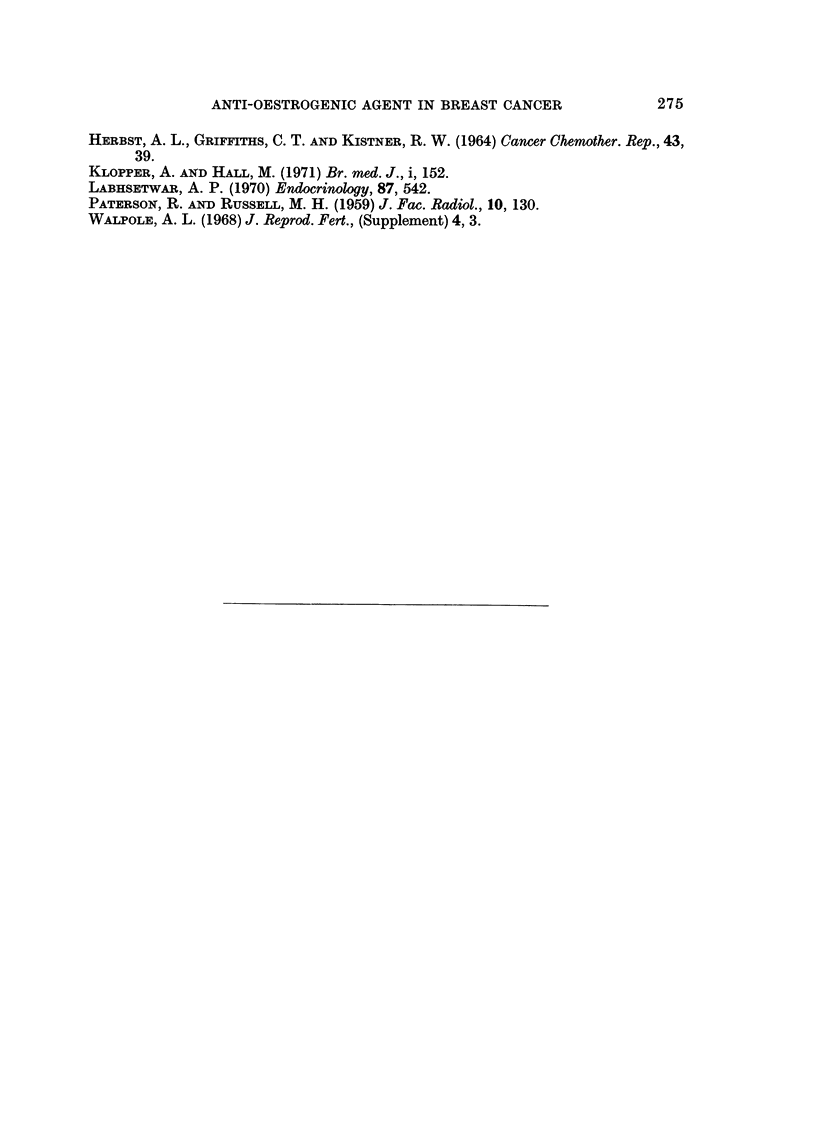# A New Anti-oestrogenic Agent in Late Breast Cancer: An Early Clinical Appraisal of ICI46474

**DOI:** 10.1038/bjc.1971.33

**Published:** 1971-06

**Authors:** M. P. Cole, C. T. A. Jones, I. D. H. Todd

## Abstract

An introductory clinical trial of the anti-oestrogenic agent IC146474 in late or recurrent carcinoma of the breast is described.

Forty-six patients have been treated, of whom 10 have shown a good response. This is of the same order as that seen with oestrogens and androgens.

The particular advantage of this drug is the low incidence of troublesome side effects.


### A NEW ANTI-OESTROGENIC AGENT IN LATE BREAST CANCER

### AN EARLY CLINICAL APPRAISAL OF ICI46474

M. P. COLE, C. T. A. JONES AND I. D. H. TODD

*From the Christie Hospital and Holt Radium Institute, Manchester M20 9BX*

Received for publication April 7, 1971

**SUMMARY.—An introductory clinical trial of the anti-oestrogenic agent ICI46474 in late or recurrent carcinoma of the breast is described.**

**Forty-six patients have been treated, of whom 10 have shown a good response. This is of the same order as that seen with oestrogens and androgens.**

**The particular advantage of this drug is the low incidence of troublesome side effects.**

ALTHOUGH the value of hormones in the palliative treatment of recurrent breast cancer is well established, clinical experience shows that only a proportion of cases respond. Laboratory tests designed to select those patients likely to benefit have produced inconclusive results and treatment has therefore been conducted on a trial and error basis.

The current practice in this hospital is to offer irradiation of the ovaries at the time of initial treatment of the breast cancer to all pre-menopausal women and to those within 3 years of the menopause. The advantage of this scheme was demonstrated by clinical trial (Paterson and Russell, 1959; Cole, 1967).

Patients with recurrence of carcinoma of the breast or metastases which are judged not suitable for radiotherapy receive hormone therapy. The younger patient within 5 years of the menopause receives androgens with or without additional cytotoxic drugs whilst the older patient 5 years or more past the menopause receives oestrogens. The group which shows the poorest response rate to hormone therapy is composed of those patients who develop their carcinoma at about the time of the menopause.

### *ICI*46474

ICI46474 is the *trans*-isomer of 1(p-beta-dimethylaminoethoxy-phenyl)-1, 2-diphenylbut-l-ene. In several, but not all mammalian species, it is a potent anti-oestrogen. Thus in low doses it opposes the action of exogenous oestrogens in cornifying the vaginal epithelium and increasing uterine weight in immature rats (Harper and Walpole, 1966). Similarly, when given to rats on the third or fourth day of pregnancy, it will prevent implantation of the fertilized ova by counteracting the augmented endogenous oestrogen release required to initiate implantation in this species (Harper and Walpole, 1967*a*, 1967*b*). In a single dose, given at the appropriate time to cycling female rats, it will delay ovulation by interfering with the oestrogen-dependent ovulatory release of luteinizing hormone (Labhsetwar, 1970). All these effects are almost certainly due to competitive interference with oestradiol uptake by specific receptors at various sites (Skidmore, unpublished data). However, the possibility that ICI46474 will also inhibit the production of oestrogen cannot be excluded on present evidence. Furthermore, very high doses of the drug have oestrogenic effects in rats. Since low doses of ICI46474 also evoke anti-oestrogenic responses in monkeys (Walpole, unpublished), it was thought probable that man would respond similarly and a number of possible clinical applications were suggested. These include the treatment of some patients with infertility due to failure of ovulation, as with clomiphene—an agent of similar chemical structure (Walpole, 1968)—and a possible contraceptive effect if oestrogen is essential for implantation of the fertilized ovum in humans. A preliminary communication on the effect of this drug in causing ovulation in secondary amenorrhoea has been published by Klopper and Hall (1971). An action on hormone dependent breast cancer was also considered and it was decided that a trial of the compound in such cases would be a suitable introduction to its clinical study.

### PATIENTS AND METHODS

All patients treated with ICI46474 had breast cancer, proved by biopsy. In every case, the extensive nature of the disease precluded a curative approach by surgery or radiotherapy. The majority of patients had already been treated with hormones or alkylating agents and all were post-menopausal. The drug was supplied in 10 mg. tablets, the dose being 1 or 2 tablets daily.

A response to the agent was accepted when there was a substantial reduction in size of soft tissue masses and/or radiological evidence of regression of pulmonary or bone metastases, together with subjective improvement, this being sustained for more than 3 months. Care was taken to ensure that no response could have been the result of treatment with, or withdrawal of, another hormone, or to any other form of coincidental treatment, particularly irradiation or antibiotic therapy. Those failing to respond to ICI46474 showed no subjective or objective improvement of any duration, but a large intermediate group was also noted. These latter patients showed subjective improvement without objective clinical or radiographical regression, or if such regression did occur, its duration was less than 3 months, or could have been attributed to another coincidental form of therapy.

The following measurements were made routinely in all patients:

haemoglobin
total and differential white cell count
platelet count
erythrocyte sedimentation rate (ESR)
serum calcium
serum inorganic phosphate
serum alkaline phosphatase
blood urea
serum alanine aminotransferase (SGPT)

In some patients, the following additional investigations were made:

determination of serum cholesterol and desmosterol levels, examination of vaginal smears and estimation of plasma oestrogen levels.

### RESULTS

Forty-six patients have received ICI46474 for longer than 3 months. Their age distribution is shown in Table I. Ten patients (22 %) have shown a clear

TABLE I.—*Patients Treated with ICI46474*

| Age (years) | 30–39 | 40–49 | 50–59 | 60–69 | 70–79 | 80–89 |
|---|---|---|---|---|---|---|
| Total number treated | 1 | 10 | 13 | 17 | 4 | 1 |
| Number responding | 0 | 4 | 1 | 3 | 2 | 0 |

response as defined above. Nineteen patients have failed to respond, whilst 17 patients have demonstrated an incomplete or indeterminate response. In 7 of the responding cases, malignant infiltration of the skin of the chest wall regressed or healing of a malignant ulcer occurred. Two patients showed radiological resolution of pulmonary metastases whilst in 1 instance, lytic bone metastases re-ossified.

A total of 19 side effects were reported and these were as follows:

| | |
|---|---|
| hot flushes | 7 |
| gastro-intestinal intolerance | 6 |
| tumour pain | 2 |
| pruritus vulvae | 1 |
| ankle oedema | 1 |
| vaginal bleeding | 1 |
| lassitude | 1 |

Many of these reactions were mild and were only admitted to on direct questioning. On only 2 occasions (4 %) has treatment been prematurely discontinued because of toxic reactions—1 patient suffered intolerable hot flushes, and the other, nausea and vomiting. This latter patient experienced similar difficulty in taking other tablets of various types. Neither patient who suffered tumour pain benefited from ICI46474.

Certain features noted during the routine blood tests deserve comment. On 4 occasions, platelet counts fell to less than 80,000/mm.$^3$ in each instance returning spontaneously to normal without discontinuing ICI46474. Three patients, enjoying a clinical response, showed a transient rise in serum alanine aminotransferase (SGPT) and it was suggested, in the absence of any additional evidence of hepatotoxicity, that this was the result of increased neoplastic cell destruction by the drug. In all responders except one, the erythrocyte sedimentation rate, if initially raised, fell to normal during treatment as the patient's general condition improved. A raised alkaline phosphatase level returned to the normal range in 2 patients. Estimation of cholesterol and desmosterol revealed normal ratios on 25 occasions.

There has been no evidence of hepatotoxicity and no hypercalcaemia. Vaginal smears showed no characteristic pattern and in these post-menopausal women, plasma oestrogen levels were too low initially to permit further useful study.

### EVALUATION

When evaluating the results with ICI46474, an unpublished trial of diethylstilboestrol against the oral androgen methylandrostenediol, conducted at this hospital, was used for purposes of comparison.

All patients entering this trial had recurrent breast cancer which had reached a stage where further surgery or radiotherapy was unlikely to be curative. Every patient was more than 5 years post-menopausal and none had previously received hormones. The patients were randomly assigned to 2 groups; 1 group received diethylstilboestrol in a dose of 5 mg. orally 3 times daily, whilst the second group was treated with methylandrostenediol in a dose of 50 mg. 4 times daily sublingually. Responses were assessed according to the criteria described above for ICI46474.

Sixty-four patients received diethylstilboestrol, of whom 16 (25 %) responded. Sixty patients received methylandrostenediol and 10 (16 %) responded. Regression of soft tissue disease accounted for the majority of responses in both groups. Thirty-six patients receiving diethylstilboestrol experienced side effects and these were of sufficient severity to terminate treatment on 12 occasions (18 %). Gastro-intestinal intolerance, fluid retention and vaginal bleeding were the commonest toxic reactions encountered. Intolerance to methylandrostenediol was observed on 17 occasions; treatment however was discontinued for this reason in only 5 instances (8 %). Fluid retention, vomiting and virilization were the most troublesome side effects in this group.

Despite the differences in the method of selection of patients who received ICI46474 as opposed to those who entered the oestrogen-androgen trial, the criteria adopted for assessment of responses were identical in both series. A limited comparison of the results obtained with the three agents has therefore been made.

The response rates to the 3 drugs are shown in Table II and the incidence of side effects in Table III.

Where diethylstilboestrol or methylandrostenediol was administered before ICI46474, the responses are summarized in Tables IV and V. No striking pattern reveals itself. In particular, there is no obvious correlation in this limited number of cases between androgen response and antioestrogen response in the same tumour.

TABLE II

| Hormone | Response rate (%) |
|---|---|
| Diethylstilboestrol | 25 (16/64) |
| Methylandrostenediol | 16 (10/60) |
| ICI46474 | 22 (10/46) |

$P > 0{\cdot}05$

TABLE III.—*Side Effects of Hormones*

| | Diethylstilboestrol | Methylandrostenediol | ICI46474 |
|---|---|---|---|
| Patients receiving hormone | 64 | 60 | 46 |
| Number of patients showing side effects | 36 (54%) | 17 (28%) | 17 (37%) |
| Drug discontinued on account of side effects | 12 (18%) | 5 (8%) | 2 (4%) |

TABLE IV.—*Previous Hormone Treatment in Patients Responding to ICI*46474

| Hormone | Response | No response |
|---|---|---|
| Diethylstilboestrol | 3 | 2 |
| Methylandrostenediol | 1 | 1 |

TABLE V.—*Previous Hormone Treatment in Patients who Failed to Respond to ICI*46474

| Hormone | Response | No response |
|---|---|---|
| Diethylstilboestrol | 3 | 9 |
| Methylandrostenediol | 5 | 7 |

Eleven of the 46 patients were treated with a dose of 10 mg. daily. Of the 11 receiving the lower dose, 4 were classed as responders.

### DISCUSSION

The evidence that ICI46474 is an oestrogen antagonist in rats and in monkeys is convincing and the likelihood that a similar effect would be seen also in man led to this trial in recurrent breast cancer. The chemically related agent clomiphene has already been used in similar cases with some evidence of response, although the mechanism of action in this situation was not conclusively proved (Herbst *et al.*, 1964).

The remission rate of 22% induced by ICI46474 in this series of patients is similar to that usually obtained with hormones. It has not been possible to confirm by laboratory investigation that ICI46474 acts directly on breast tumours by oestrogen antagonism, owing to the low levels of circulating oestrogens found in these patients, but the occurrence, or exacerbation, of hot flushes in some treated patients suggests that this is the likely explanation. This particular side effect was not observed when using diethylstilboestrol or methylandrostanediol.

Although the response rates in the oestrogen-androgen trial compare closely with that for ICI46474, the incidence of side effects of sufficient severity to terminate treatment with the latter is lower, particularly when compared with diethylstilboestrol. Gastro-intestinal intolerance and fluid retention are less marked following the anti-oestrogen than diethylstilboestrol, whilst the virilizing effect of androgens has not been seen with ICI46474. The occasional fall in the platelet count is difficult to explain, although 3 of the 4 patients had received alkylating agents before ICI46474. The lower toxicity of the anti-oestrogen seems to constitute its main advantage over other hormones.

Four of the 10 responses to ICI46474 occurred in the fifth decade, within 3 years of the menopause, a group which in the past has usually been particularly insensitive to hormone therapy.

There is no convincing correlation between androgen responsiveness and anti-oestrogen responsiveness of the same tumour. Cases relapsing after a beneficial response to a standard hormone may be expected in some cases to respond later to ICI46474 in view of its different mode of action.

We are grateful to Dr. R. T. Rouse and Dr. A. L. Walpole for their advice and to Imperial Chemical Industries Limited for supplying ICI46474.

### REFERENCES

COLE, M. P.—(1967) in ' Prognostic Factors in Breast Cancer, ' edited by A. P. M. Forrest and P. B. Kunkler. Edinburgh and London (E. and S. Livingston Ltd) p. 46.

HARPER, M. J. K. AND WALPOLE, A. L.—(1966) *Nature, Lond.*, **212**, 87.—(1967*a*) *J. Endocr.*, **37**, 83.—(1967*b*) *J. Reprod. Fert.*, **13**, 101.

Herbst, A. L., Griffiths, C. T. and Kistner, R. W. (1964) *Cancer Chemother. Rep.*, **43**, 39.
Klopper, A. and Hall, M. (1971) *Br. med. J.*, i, 152.
Labhsetwar, A. P. (1970) *Endocrinology*, **87**, 542.
Paterson, R. and Russell, M. H. (1959) *J. Fac. Radiol.*, **10**, 130.
Walpole, A. L. (1968) *J. Reprod. Fert.*, (Supplement) **4**, 3.